# The Relationship between Frontal, Axial Leg Alignment, and Ankle Joint Line Orientation—a Radiographic Analysis of Healthy Subjects

**DOI:** 10.1111/os.13567

**Published:** 2022-11-09

**Authors:** Sandro Hodel, Nicola Cavalcanti, Sandro Fucentese, Lazaros Vlachopoulos, Arnd Viehöfer, Stephan Wirth

**Affiliations:** ^1^ Department of Orthopaedics Balgrist University Hospital, University of Zurich Zurich Switzerland; ^2^ Balgrist University Hospital University of Zurich Zürich Switzerland

**Keywords:** Alignment, Ankle, Foot, Joint Line

## Abstract

**Objective:**

Ankle joint line orientation (AJLO) is influenced by the subtalar foot and frontal leg alignment. However, the influence of axial leg alignment on AJLO remains unclear. The study aimed to analyze the influence of frontal, axial leg alignment on AJLO in healthy subjects.

**Methods:**

Thirty healthy subjects (60 legs) without prior surgery underwent standing biplanar long leg radiograph (LLR) between 2016 and 2020. AJLO was measured in standing long‐leg radiographs relative to the ground. Meary's angle and calcaneal pitch were measured. Hip‐knee‐ankle angle (HKA), femoral antetorsion, and tibial torsion were assessed with SterEOS software (EOS Imaging, Paris, France). LLR was acquired with the feet directing straight anteriorly, which corresponds to a neutral foot progression angle (FPA). The influence of subtalar, frontal, and axial alignment on AJLO was analyzed in a multiple regression model.

**Results:**

An increase in knee valgus increased relative valgus AJLO by 0.5° (95% CI: 0.2° to 0.7°) per 1° (*P* < 0.001). A decrease in femoral antetorsion increased relative valgus AJLO by 0.2° (95% CI: 0.1° to 0.2°) per 1° (*P* < 0.001), whereas Meary's angle and calcaneal pitch did not influence AJLO.

**Conclusion:**

A link between frontal, axial leg alignment, and AJLO could be demonstrated, indicating that a valgus leg alignment and relative femoral retrotorsion are associated with an increase of valgus AJLO in healthy subjects when placing their feet in a neutral position. Alteration of the frontal, or rotational profile after realignment surgery or by implant positioning might influence the AJLO, when the FPA is kept constant.

## Graphical abstract

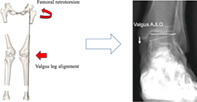

## Introduction

In total ankle arthroplasty (TAA), concomitant realignment procedures are well‐established to align the components neutrally. The goal to establish a neutral AJLO favors equal load‐bearing, decreases wear, and is likely to influence long‐term survivorship of TAA.^1^, ^2^ Most common realignment procedures include hindfoot osteotomies and distal tibial osteotomies at the site of the deformity.^3^, ^4^ However, in addition to distal tibial or hindfoot deformities, nearly 30% of patients scheduled for TAA demonstrated a significant concomitant frontal malalignment of the leg.^5^

Therefore, the influence of leg alignment, especially varus/valgus deformities, on AJLO has gained attention following osteotomies around the knee or total knee arthroplasty (TKA).^6^, ^7^ A correction of leg alignment demonstrated a close relationship to the correction of AJLO and the mechanical load shift in the ankle,^8^ underlining the importance of evaluating the whole kinematic chain of the lower extremities under weight‐bearing conditions. Previous studies mainly focused on the frontal leg alignment and interconnected relationship of the hip, knee, and ankle, neglecting the importance of axial alignment.^7^, ^8^, ^9^ This focus solely on frontal alignment seems insufficient, as it is most likely not only frontal but also axial leg alignment and sagittal pelvic orientation, which influence the kinematic chain under weight‐bearing load and, therefore, AJLO. This is especially of clinical relevance, when evaluating patients with concomitant joint disorders at the lower extremities either for a TAA or an axial realignment of the lower extremity. The planning of corrective osteotomies and implant positioning requires a more profound understanding of the interconnection of frontal and axial alignment. However, previous studies often lacked advanced imaging studies, including axial leg or pelvic alignment.

The aim of the study was^1^ to analyze the influence of frontal and axial leg alignment on AJLO in healthy subjects and to investigate the influence of^2^ pelvic and subtalar alignment on AJLO in a radiographic assessment using long‐leg‐radiographs of healthy subjects.

## Methods

### Study Cohort

Thirty healthy volunteers (60 lower legs) underwent prospective clinical and radiographic analysis at the authors’ institution between 2016 and 2020 after ethical approval and trial registration (KEK‐Nr: 2016–00410). Inclusion criteria comprised: (i) patients without prior surgery or symptoms of the lower extremities; and (ii) age > 18 years. Absence of symptoms or prior surgery at the lower extremities was confirmed by an orthopedic surgeon who performed a clinical examination in all candidates. Exclusion criteria were missing informed consent. Sixteen female subjects and 14 male subjects (60 legs) with a mean age of 27.1 standard deviation *±* 10.0 years (range 20–67 years) were included (Table. 1). No patients needed to be excluded.

**TABLE 1:** Demographic characteristics

Demograph	30 subjects (60 legs)
Age (years)	27.1 ± 10 (20 to 67)
Male	14 (46.7)
Female	16 (53.3)
BMI (kg/m^2^)	21.3 ± 2.2 (17.0 to 26.2)

*Note*: Data reported as mean ± SD (range) or counts (%) if not stated otherwise

Abbreviation: BMI: Body‐mass index.

### Radiographic Analysis of the Foot

**Fig. 1:**
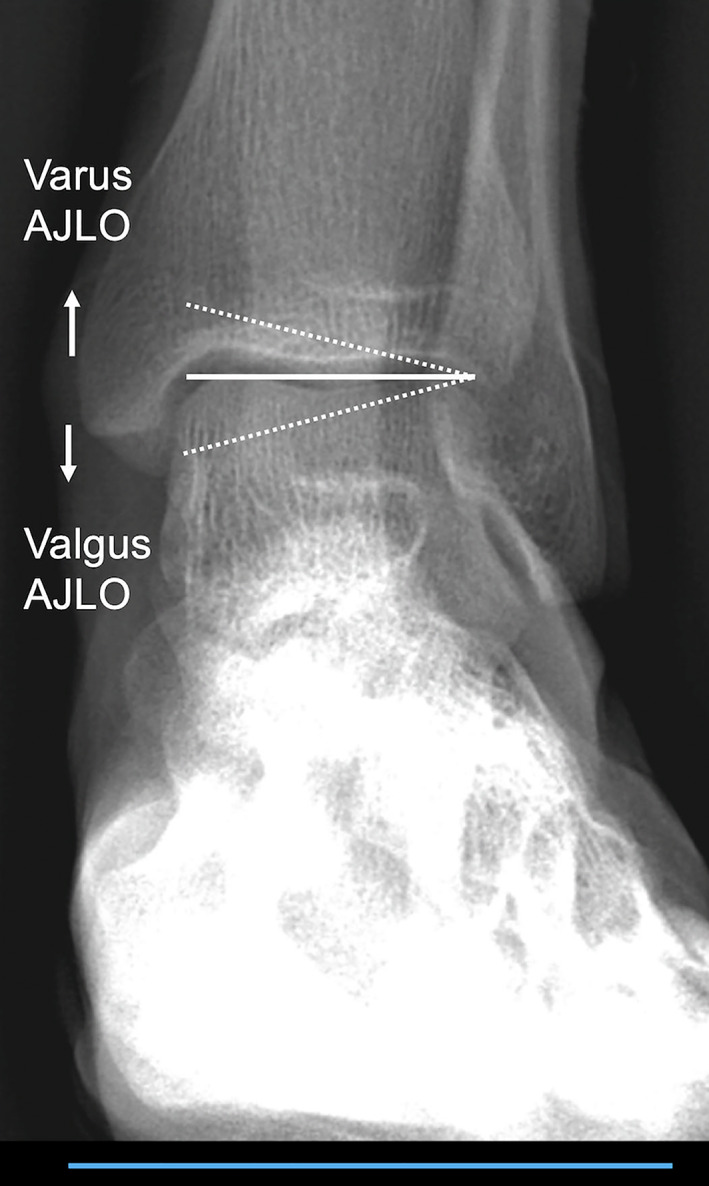
Ankle joint line orientation (AJLO) was measured between a line connecting the most proximal point of the medial and lateral talar dome (white line) and the ground (blue line) (°). A medial downward slope was defined as a relative valgus AJLO (dotted line) and a medial upward slope as a varus AJLO (dotted line).

AJLO was measured relative to the ground in standing long‐leg radiographs, a medial downward slope indicating a relative valgus AJLO (Fig. 1). Additionally, subtalar foot alignment was assessed by Meary's angle^10^ and calcaneal pitch^11^ in standing long‐leg radiographs.

Two blinded observers performed all foot radiographic measurements using a Picture Archiving and Communication System (PACS) (MERLIN version 5.8.1.1980, Phoenix PACS GmbH ©, Freiburg im Breisgau, Germany).

### Radiographic Analysis of Leg Alignment and Pelvic Orientation

Radiographic assessment of leg and pelvic alignment included biplanar standing long‐leg radiographs EOS (EOS Imaging, Paris, France). All subjects were instructed to stand with their feet aligned neutrally to the frontal plane, and a Wii balance board (Nintendo ©) was used to ensure even load distribution during image acquisition. All subjects were instructed to place their feet straight directing anterior which corresponds to a neutral foot progression angle (FPA), a centered patella then confirmed this. Hip‐knee‐ankle angle (HKA), femoral antetorsion, tibial torsion, and pelvic tilt were measured using SterEOS software (EOS Imaging, Paris, France). Three‐dimensional (3D) models of the femur and tibia were semi‐automatically registered onto bony landmarks of the biplanar long leg radiograph as described by Buck *et al*.^12^ The software performed an automatic measurement procedure of HKA, femoral antetorsion, tibial torsion, and pelvic tilt and has previously been reported with a high accuracy^8^, ^12^ and reliability.^13^, ^14^ HKA (°) was measured in the frontal plane between a line formed by the hip center and the center of the tibial eminences and a line connecting the tibial eminences and the center of the tibial plafond. Negative values correspond to a relative valgus alignment. Femoral antetorsion (°) was measured between a line defined by the hip center and the center of the femoral neck and a line defined by the posterior distal femoral condyles as described by Hernandez *et al*.^15^ Negative values indicate a relative retrotorsion of the femoral neck. Tibial torsion (°) was measured between a line defined by the posterior proximal tibial cortex and a line defined by the medial and lateral malleolus as described by Goutallier *et al*.^16^ Pelvic tilt was measured in the sagittal plane according to Legaye *et al*., and a decreasing value indicates a more anterior tilting of the pelvis.^17^

### Statistics

*A priori* power analysis (*α* = 0.05, power level *β* = 0.80) revealed a minimum sample size of *n* = 53 to analyze the influence of four predictors on AJLO (dependent variable) in a linear regression model with a weak to medium effect size (Cohen's *f* ^2^ = 0.25). The power analysis was conducted using G*Power (version 3.1; Franz Faull, Universität Kiel, Germany).

The normal distribution of the data was tested with Shapiro–Wilk's test. Data are reported as mean ± standard deviation (SD) or counts (percentages). Inter‐reader reliability was calculated using intraclass correlation coefficients (ICC) assuming a two‐way mixed‐effect and complete agreement and graded according to Fleiss^18^ (>0.75 indicating excellent reliability). The means of both readers were used for further analysis. Gender differences of AJLO were analyzed with a Mann–Whitney‐U test. A bivariate correlation between age, sex, Meary's angle, calcaneal pitch, HKA, femoral antetorsion, tibial torsion, pelvic tilt, and AJLO was performed using Spearman's rank correlation or Pearson's R as appropriate. All factors that demonstrated a minimum correlation coefficient of 0.3 were further included in a multiple regression analysis. The absence of multicollinearity was tested by the variance inflation factor (VIF). Multicollinearity was defined if VIF ≧10. The fit of the regression model was reported as *R* ^2^. Change of AJLO (°) (95% confidence interval (CI)) for each variable was reported. The significance was set <0.05. Data were analyzed with SPSS version 26 (SPSS Inc., Chicago, IL, USA).

## Results

### Radiographic Assessment and Reliability Testing

Radiographic characteristics of foot, leg and pelvic alignment are summarized in Table. 2. Inter‐reader reliability for AJLO, Meary's angle and calcaneal pitch was 0.97 (95% CI: 0.95–0.98), 0.96 (95% CI: 0.93–0.97) and 0.93 (95% CI: 0.86–0.96) (all *P* < 0.001), indicating excellent reliability.

**TABLE 2:** Radiographic analysis

AJLO (°)	0.5 ± 4.4 (−11.7 to 10.0)
Meary's angle (°)	−2.3 ± 6.6 (−16.6 to 9.9)
Calcaneal pitch (°)	23.0 ± 4.7 (12.7 to 33.4)
HKA (°)	1.7 ± 3.7 (−8.0 to 9.0)
Femoral antetorsion (°)	17.1 ± 11.2 (−7.0 to 52.0)
Tibial torsion (°)	31.9 ± 6.7 (13.0 to 45.0)
Pelvic tilt (°)	13.4. ± 7.5 (−2.0 to 29.0)

*Notes*: Ankle joint line orientation (AJLO) (+ varus AJLO/‐ valgus AJLO). Meary's angle (+ pes cavus/− pes planus) HKA: Hip‐knee‐ankle angle (+ varus/− valgus). Femoral antetorsion (+ antetorsion/‐ retrotorsion). Data reported as mean ± SD (range) or counts (%) if not stated otherwise

Mean AJLO was 0.5 ± 4.4°, independent of gender (*P* = 0.568).

### Influence of Frontal and Axial Leg Alignment on Ankle Joint Line Orientation

The univariate analysis yielded a significant correlation between HKA and AJLO and femoral antetorsion and AJLO (both *P* < 0.001) (Table. 3).

**TABLE 3:** Influence of demographics, foot, leg, and pelvic alignment on ankle joint line orientation

Predictor variable	Univariate correlation with AJLO (°) Correlation coefficient (*p*‐value)	Multivariate regression: Change in AJLO (°) (95% CI; *p*‐value)
Gender	0.07 (0.573)*	NA
Age (years)	0.15 (0.259)*	NA
BMI (kg/m^2^)	−0.22 (0.110)	NA
Meary's angle (°)	0.13 (0.313)	NA
Calcaneal pitch (°)	−0.11 (0.420)	NA
HKA (°)	0.46 **(<0.001)**	0.5 (0.2 to 0.7; **<0.001)**
Femoral antetorsion (°)	0.46 (<0.001)*	0.2 (0.1 to 0.2; **<0.001)**
Tibial torsion (°)	0.04 (0.753)	NA
Pelvic tilt (°)	0.19 (0.149)	NA

*Note*: Significant factors in univariate analysis with a minimum correlation coefficient of 0.3 (*Spearman's rank, remaining Pearson's R, middle row) included in multiple regression model (right row). Overall linear regression model fit explained 37% of variance AJLO by HKA and femoral antetorsion (*R* ^2^ = 0.37; *P* < 0.001). Significant factors marked bold (*P* < 0.05).

Abbreviations: BMI, Body‐mass index; HKA, Hip‐knee‐ankle angle; NA, Not applicable.

**Fig. 2:**
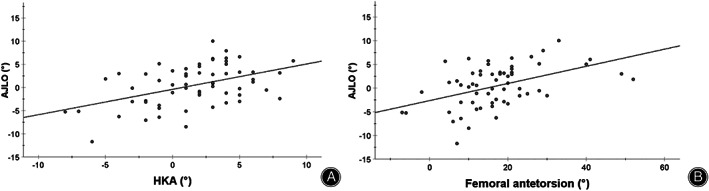
Relationship between frontal, axial leg alignment, and ankle joint line orientation. (A): Scatterplot depicts the relationship between ankle joint line orientation (AJLO) and hip‐knee‐ankle angle (HKA). (B): Scatterplot represents the relationship between AJLO and femoral antetorsion.

In multivariate regression analysis, an increase in knee valgus was associated with an increase of valgus AJLO by 0.5° (95% CI: 0.2° to 0.7°) per 1° (*P* < 0.001). A decrease in femoral antetorsion was associated with an increase of valgus AJLO by 0.2° (95% CI: 0.1° to 0.2°) per 1° (*P* < 0.001). Meary's angle and calcaneal pitch did not influence AJLO (Table. 3, Fig. 2). Overall linear regression model fit explained 37% of the variance in AJLO (*R* ^2^ = 0.37; *P* < 0.001) by HKA and femoral antetorsion. All analyzed factors are listed in Table. 3.

## Discussion

The most important finding of our study is that frontal leg alignment and axial leg alignment influence AJLO. An increase in valgus alignment and femoral retrotorsion favored an increasing valgus AJLO in healthy subjects when their feet are placed in a neutral anterior position independent of subtalar alignment.

### Influence of Frontal Alignment on Ankle Joint Line Orientation

Assessment of concomitant deformity at the distal tibia or hindfoot and its effect on the load distribution in the ankle joint has been recognized previously in foot and ankle surgery.^3^, ^19^ In addition, the influence of frontal leg alignment on AJLO after high tibial osteotomies or TKA has recently been described,^6^, ^20^ and the relationship between frontal leg alignment and AJLO could be reproduced in our cohort. This is in line with a previous study by Khamis and Yizhar that correlated foot hyperpronation with valgus leg alignment.^21^ According to our knowledge, this is the first study yet to analyze the influence of axial leg alignment on AJLO.

### Influence of Axial Alignment on Ankle Joint Line Orientation

Concerning the simplistic model of a tripod of the foot, axial alignment seems logical to influence the calcaneal and, therefore, hindfoot position and AJLO. As seen in a long‐leg radiograph, straight anteriorly placement of the foot results in a relative internal leg rotation in a patient with anatomical femoral retrotorsion, placing the hindfoot further laterally and favoring an increase in valgus AJLO. In contrast, femoral antetorsion and in‐toeing leads to a hindfoot medialization when placing the foot straight forward and increases varus AJLO, underlining the interconnection between frontal axial leg alignment and AJLO. This interconnection should be accounted for during clinical evaluation of patients scheduled for foot and ankle realignment procedures or TAA.

### Clinical Relevance in Total Ankle Arthroplasty and Axial Realignment Procedures

The clinical relevance of the reported findings are that identifying patients with concomitant maltorsion of the lower extremities or an excessive FPA could improve implant positioning and functional outcome after TAA. Accounting for these factors might favor a more customized approach for TAA positioning. For future studies the role of axial alignment of the lower extremities and gait analysis on functional outcome after TAA should be investigated, especially as a relationship between the torsional profile and the FPA has been established before.^22^ Especially regarding the long‐term survivorship after TAA, a well‐balanced TAA is highly desirable to avoid uneven load distribution and, polyethylene wear^23^ and ligament imbalance. To improve accuracy and patient outcome following TAA, several navigation tools have been described as patient‐specific instrumentation^24^ or computer‐assisted navigation.^25^

This relationship is of equal clinical importance in patients scheduled to correct excessive femoral malrotation or frontal realignment procedures with simultaneous symptoms, ligament instabilities, or signs of degeneration at the ankle joint. Unintentional created obliquity of the AJLO after a corrective osteotomy could potentially deteriorate a pre‐existing ankle disorder. Patients scheduled for torsional realignment >20° are especially at risk for coronal malalignment^26^ and should be screened for concomitant ankle pathologies. Furthermore, the extent of the planned surgical correction should be evaluated individually based on the patient's symptoms and adjacent joint disorders.

How patients with varying axial leg alignment place their feet during walking is most likely dependent on concomitant pathologies at the knee or hip and cannot be answered based on our static radiographic analysis of healthy subjects. Nevertheless, the identified relationship between axial leg alignment and AJLO builds the base for future radiographic or functional analysis in patients undergoing foot and ankle surgery or leg realignment procedures.

### Strenghts and Limitations

The investigated cohort is well balanced regarding leg alignment and gender and therefore representative of an average population. The radiographic analysis was performed blind and demonstrated excellent reliability among the readers.

Several limitations have to be considered when interpreting our findings. The outcome measurement was limited to a static radiological analysis and did not consider dynamic motion patterns during activities. However, the presented findings identified axial leg alignment as a significant factor for AJLO, warranting further functional and radiographic research.

The decision to include SterEOS imaging, based on biplanar radiographs, most likely influences the reported leg alignment measurements but has been reported with excellent accuracy and reliability in previous studies.^13^, ^14^ The assessment of Meary's angle and the calcaneal pitch was not initially described in long‐leg radiographs and, therefore, may have altered our reported means. Nevertheless, both measurements yielded excellent reliability. Additionally, hindfoot varus/valgus alignment could not be measured in our cohort but is a contributing factor influencing AJLO. However, in a healthy cohort without symptoms at the feet, excessive hindfoot varus or valgus is unlikely to be present and most likely did not significantly influence our findings.

### Conclusion

A link between frontal, axial leg alignment, and AJLO could be demonstrated in this radiographic analysis. A valgus leg alignment and relative femoral retrotorsion lead to an increase of valgus AJLO in healthy subjects when placing their feet in a neutral position. The alteration of the frontal, or rotational profile after realignment surgery or by implant positioning needs to be considered in patients with multiple joint disorders of the lower extremities.

## Author Contributions

Sandro Hodel conceived the design of the study. Sandro Hodel and Nicola Cavalcanti performed the data acquisition. Sandro Hodel performed the statistical analysis, prepared the graphics and drafted the manuscript. Sandro Hodel, Arnd Viehöfer, Stephan Wirth and Lazaros Vlachopoulos revised the manuscript critically. Stephan Wirth, Sandro Fucentese helped with the study design and supervision of the experiments. All authors read and approved the final manuscript. All authors listed meet the authorship criteria according to the latest guidelines of the International Committee of Medical Journal Editors and approved the final manuscript.
